# Feasibility of an Innovative Rehabilitation Program Adapted for the Post-acute Nursing Home Setting

**DOI:** 10.1093/geroni/igab046.3396

**Published:** 2021-12-17

**Authors:** Addie Middleton, Jane Driver, Marcus Ruopp, Lindsay Lefers, Jessica Rawlins, Rebekah Harris, Jonathan Bean

**Affiliations:** 1 VA Boston, Boston, Massachusetts, United States; 2 VA Boston Healthcare System, Brockton, Massachusetts, United States; 3 VA Boston Healthcare System, VA Boston Healthcare System, Massachusetts, United States

## Abstract

Live Long Walk Strong is a rehabilitation program that produces large clinically meaningful improvements in mobility when implemented as an outpatient program for older adults. We adapted Live Long Walk Strong for the post-acute nursing home setting within the Veterans Health Administration as a clinical demonstration project. The adapted version includes novel elements and bridges the inpatient stay and three months post-discharge. The inpatient phase focuses on maximizing functional recovery and includes activities focused on timing and coordination of gait, leg strength and power, and trunk muscle endurance. The care transition and virtual (telehealth) post-discharge phase focuses on case management and engagement in physical activity programs. Coaching and behavior change are a consistent focus throughout the program. To date, 13 Veterans (mean age 67.9, SD 11.7 years) have completed the inpatient phase, and of those Veterans, six have completed the entire program, five are still active, one was lost to follow-up, and one was rehospitalized. The program demonstrates feasibility, 91% of all inpatient sessions and 81% of all post-discharge sessions were completed. Regarding preliminary efficacy, 83% of Veterans who completed the program exceeded the minimal detectable change score (4 points) on the Activity Measure for Post-Acute Care (AM-PAC) Mobility scale from program enrollment to completion (mean change 6.5, SD 6.9 points). Based on findings from this clinical demonstration project, the program is feasible. However, future research is needed to further examine the program’s impact on mobility and other outcomes important to older Veterans receiving post-acute nursing home care.

